# SERS-based microdroplet platform for high-throughput screening of *Escherichia coli* strains for the efficient biosynthesis of D-phenyllactic acid

**DOI:** 10.3389/fbioe.2024.1470830

**Published:** 2024-09-20

**Authors:** Lin Hu, Ruoshi Luo, Dan Wang, Fanzhen Lin, Kaixing Xiao, Yaqi Kang

**Affiliations:** Department of Chemical Engineering, School of Chemistry and Chemical Engineering, Chongqing University, Chongqing, China

**Keywords:** D-phenyllactic acid, surface-enhanced Raman spectroscopy, microdroplet screening, directed evolution, molecular docking

## Abstract

D-Phenyllactic acid (D-PLA) is a potent antimicrobial typically synthesized through chemical methods. However, due to the complexity and large pollution of these reactions, a simpler and more eco-friendly approach was needed. In this study, a strain for D-PLA biosynthesis was constructed, but the efficiency was restricted by the activity of D-lactate dehydrogenase (DLDH). To address this issue, a DLDH mutant library was constructed and the Surface-Enhanced Raman Spectroscopy (SERS) was employed for the precise quantification of D-PLA at the single-cell level. The TB24 mutant exhibited a significant improvement in D-PLA productivity and a 23.03-fold increase in enzymatic activity, which was attributed to the enhanced hydrogen bonding and increased hydrophobicity within the substrate-binding pocket. By implementing multi-level optimization strategies, including the co-expression of glycerol dehydrogenase (GlyDH) with DLDH, chassis cell replacement, and RBS engineering, a significant increase in D-PLA yields was achieved, reaching 128.4 g/L. This study underscores the effectiveness of SERS-based microdroplet high-throughput screening (HTS) in identifying superior mutant enzymes and offers a strategy for large-scale D-PLA biotransformation.

## 1 Introduction

Phenyllactic acid (PLA) is a natural organic acid found in various foods, such as honey, milk, cheese, pickles, and sourdough (Behera et al., 2020). PLA serves as a monomer for the biodegradable material polyphenyllactic acid and exists naturelly in two enantiomeric forms (Kawaguchi et al., 2017): D- and L-PLA. D-PLA possesses superior antibacterial potential and exhibits a broad range of inhibitory effects on both gram-negative and gram-positive bacteria, as well as fungi (Mu et al., 2012a). It is also commonly utilized as a safe and effective biological preservative (Liu et al., 2020). Consequently, D-PLA is employed in various fermented food products. Furthermore, D-PLA is regarded as a promising candidate for next-generation pharmaceuticals, with potential applications in treating a range of conditions, such as colitis and diabetes (Zhou et al., 2021). In addition, D-PLA is used as a feed additive (Rajanikar et al., 2021) and is incorporated into hydrogel formulations (Sun et al., 2019). Recent researches indicated that D-PLA can be synthesized chemically and biologically. However, chemical synthesis presents inherent challenges, such as stringent conditions, extensive by-product generation, and process complexity (Costa et al., 2020). The eco-friendly and catalytically efficient biosynthesis of D-PLA from phenylalanine (Phe) or phenylpyruvate (PPA) has garnered interest, offering distinct advantages over chemical synthesis methods (Zhou Y. et al., 2024).

Due to its excellent properties, the biosynthesis of D-PLA has received widespread attention in recent years. The biological production of D-PLA can be categorized into three methods: lactic acid bacteria fermentation (Lipinska-Zubrycka et al., 2020), single-enzyme whole-cell catalysis (Zhou et al., 2018), and multi-enzyme whole-cell catalysis (Wu et al., 2023). Despite numerous reports on D-PLA biosynthesis by lactic acid bacteria (Lavermicocca et al., 2000; Schmidt et al., 2018; Guan et al., 2019), the concurrent production of by-products complicates downstream separation processes, which impedes industrial scalability (Mu et al., 2012b). By employing *Escherichia coli* overexpressing DLDH (D-lactate dehydrogenase) and culturing it at 170 rpm and 37°C for 12 h, 18.21 g/L D-PLA was synthesized from 10 g/L PPA. A yield of 90.49% and 2.49 g/(L·h) productivity were achieved by this process, respectively (Zhou et al., 2018). However, there is an oversight regarding the requirement for the cofactor NADH, which is essential for the conversion of PPA to D-PLA. To reduce the high costs associated with NADH supplementation, researchers have engineered *E. coli* strains capable of utilizing co-substrates such as glycerol (Qin et al., 2021), glucose (Zhu et al., 2017a), and formic acid (Zhu et al., 2017b) for the NADH cofactor generation. Formic acid and glucose are key substrates in generating NADH for D-PLA synthesis. Yet, the conversion of formic acid to CO_2_ by FDH (formate dehydrogenase) and glucose to gluconic acid by GDH (glucose dehydrogenase) lead to the by-products that challenge carbon retention and purification efficiency. In contrast, GlyDH (glycerol dehydrogenase) selectively converts glycerol into the versatile dihydroxyacetone, presenting a more sustainable approach (Qin et al., 2021). Advances in D-PLA biocatalysis are currently limited by the high costs of substrates, such as PPA. A yield of 72.14% was achieved by synthesizing 10.02 g/L of D-PLA from 13.89 g/L of Phe through the co-expression of L-AAD (L-amino acid deaminase), D-LDH, and FDH in *E. coli* (Zheng et al., 2018). In summary, the whole-cell biosynthesis of D-PLA encounters several challenges, including the provision of the essential cofactor NADH, the economic feasibility of substrate costs, and the catalytic inefficiency of the enzymes involved.

Natural enzyme activities frequently fail to meet the requirements of artificially engineered metabolic pathways, necessitating the development of strategies to enhance their catalytic efficacy. Numerous studies have shown that computer-aided semi-rational design is a powerful tool for enhancing enzyme catalytic activity (Chen et al., 2020; Song et al., 2023). However, the probability of beneficial mutations is too low (<10^−5^). The efficiency of conventional screening methods is hampered by a low throughput, resulting in high costs and prolonged screening times to identify a large number of beneficial mutants (Xia et al., 2021). Therefore, the development of rapid screening methods for microbial strains and enzyme mutants is essential for improving productivity (Sarnaik et al., 2020). Various high-throughput screening (HTS) strategies have been established (Zeng et al., 2020). A digital SERS microfluidic chip has been proposed, which enables the high-precision quantification of target microorganisms through the enumeration of characteristic SERS signals, using yeast as a model detection target (Wen et al., 2024). Additionally, a sensitive, label-free SERS-based sensor for COVID-19 was developed, achieving an attomole-level detection limit with high accuracy. This sensor utilizes recombinant trimeric spike proteins and is applicable to both wild-type and variant strains of the virus (Park et al., 2023). Researchers have developed SERS techniques that offer distinct spectral and aptamer signature bands. This facilitates the elucidation of key binding sites post-SELEX (systematic evolution of ligands via exponential enrichment), guiding aptamer optimization and assessing ligand affinity, thereby yielding high-affinity aptamers (Liu et al., 2022). To date, no HTS strategy for the biosynthesis of D-PLA has been documented. SERS has emerged as a powerful tool in HTS, capable of detecting a wide array of substances with distinctive and highly sensitive molecular vibrational signatures (Yang et al., 2023). Therefore, constructing a Raman spectroscopy-based microdroplet screening method will be beneficial for enhancing D-PLA yields. This novel approach can address the limitations of traditional modification processes and aim to identify superior mutant enzymes through an accelerated screening process.

**FIGURE 1:**
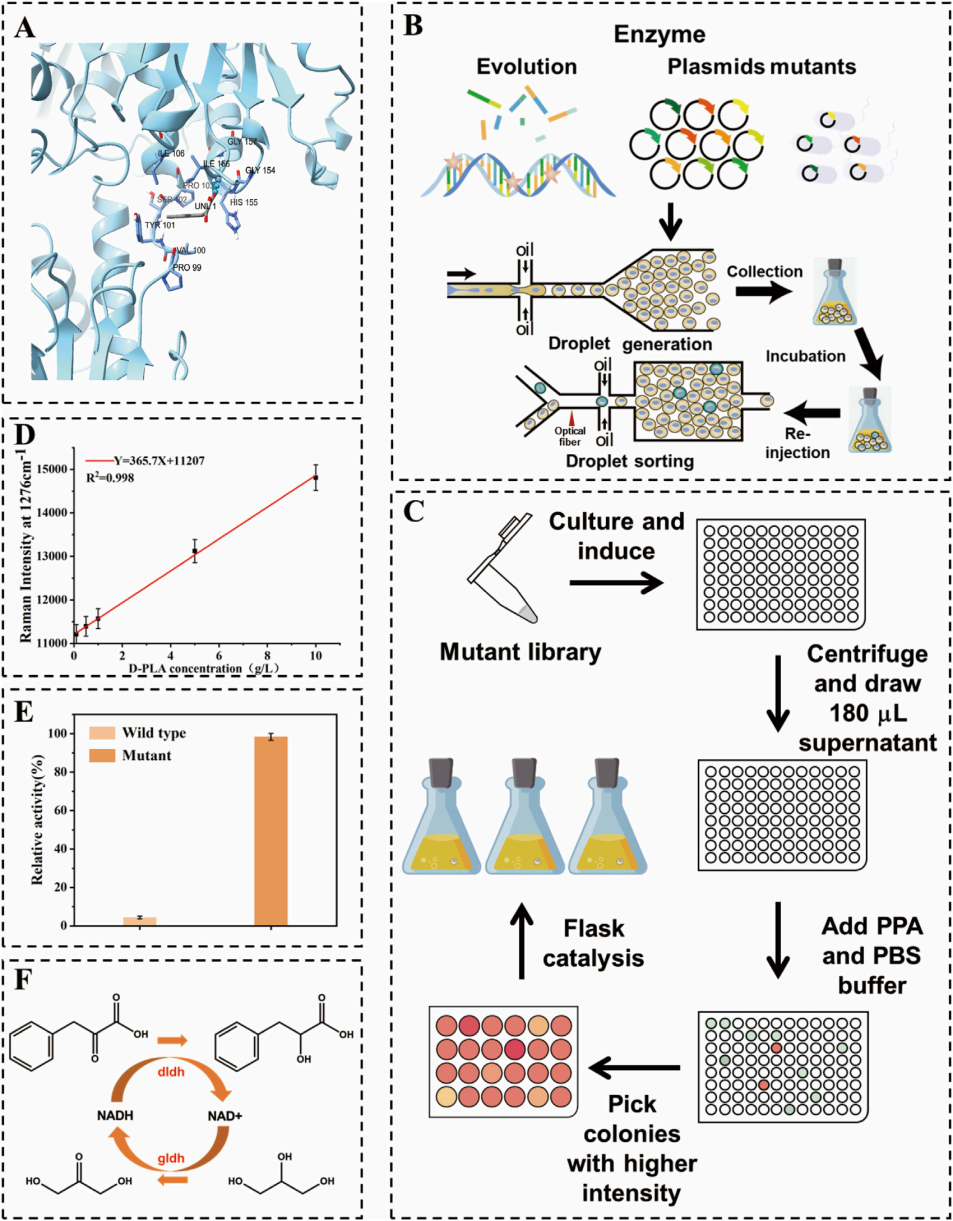
Schematic diagram of high-throughput droplet sorting for *dldh* directed evolution. **(A)** Schematic representation of DLDH docking with substrate **(B)** Construction of *dldh* mutant libraries as well as single-bacteria encapsulation, incubation, re-injection, and sorting in droplets **(C)** Validation of superior mutants using 24-well microtiter plates, 96-well microtiter plates, and shaker flasks. **(D)** Raman intensity at 1,276 cm^−1^ versus D-PLA concentration used in this study. **(E)** Comparison of enzyme activity between the wild type and the optimal mutant **(F)** Diagram of the D-PLA synthesis pathway used in this study.

A high-throughput SERS-based microdroplet sorting system was developed to enhance enzyme efficiency and D-PLA yield in whole-cell catalytic synthesis. This system enabled the rapid identification of high-yielding D-PLA strains through unlabeled screening, overcoming challenges associated with traditional modification methods. Beneficial mutants were selected from a random mutagenesis library and validated through microtiter plate and shake flask cultures. The co-expression of DLDH and GLyDH in *E. coli*, optimized with a robust strain switch from BL21 (DE3) to MG1655, facilitated D-PLA synthesis. The response path of this study is shown in Figure 1F. Enzyme expression levels were further refined by RBS optimization, thereby maximizing D-PLA production. The process was successfully scaled up to a 5 L bioreactor, confirming industrial scalability. Our findings underscore the metabolic potential of the engineered strain and the utility of the SERS platform in high-throughput strain selection for the production of valuable chemicals.

## 2 Materials and methods

### 2.1 Chemicals, strains, and media

Reagents such as sodium phenylpyruvate (PPA) and kanamycin were procured from Sangon Biotech (Shanghai, China) Co., Ltd. Tryptone and yeast extracts were purchased from Oxford Limited, United Kingdom. For molecular biology applications, PrimeSTAR HS DNA polymerase, QuickCut™ restrictive endonucleases, PCR reagents, loading buffers for nucleic acid electrophoresis, and SDS-PAGE, as well as DNA and protein markers, were all purchased from Takara Biotechnology (Dalian, China) Co., Ltd. The plasmid preparation kit was purchased from Sangon Biotech (Shanghai, China). The gel recovery kit was purchased from Zhuangmeng Biogene Technology Co., Ltd. (Beijing, China). DNA sequencing services were conducted by Tsingke Co., Ltd. (Beijing, China). Unless otherwise specified, all other reagents and chemicals used in this study were sourced from general commercial suppliers and are suitable for direct use without further purification. The host strains and vectors employed, including *E. coli* BL21 (DE3), *E. coli* MG1655 and pET-28a (+), were specifically procured from Novagen (Shanghai, China) for recombinant protein expression.

Luria-Bertani (LB) medium (pH 7.0) was prepared by dissolving 10.0 g of tryptone, 5.0 g of yeast extract and 10.0 g of NaCl in 1.0 L of distilled water. For the preparation of LB solid medium, 16.0 g of agar was added to the aforementioned solution, followed by sterilization at 121°C for 20 min. Unless otherwise stated, all other reagents and chemicals utilized in this study were sourced from standard commercial suppliers and used without additional purification.

### 2.2 Construction of random mutation library

In this study, a mutant library of *dldh* was created utilizing a random mutation kit with the pET28a-*dldh* plasmid serving as a template. Initially, a 20 µL PCR pilot experiment determined that the optimal amount of StarMut Enhancer to be added was 3 µL. This quantity allowed for precise control over the base mutation rate, aiming for a specific mutation frequency of 2.8 mutations per kilobase. Following this optimization, a 50 µL PCR protocol was used to successfully generate a mutant library for the *dldh* gene. The plasmid pET28a-*dldh* was obtained from the laboratory depository. The term dldh* denotes the *dldh* gene with mutations at the A174Q and V331R loci. The library was generated by introducing base mutations during the amplification process, which utilized an error-prone, heat-resistant DNA polymerase. The resulting error-prone PCR product was subsequently inserted into a cleaved vector, as depicted in Figure 1B. The mutant library was subsequently introduced into BL21 (DE3) cells for protein expression. A positive screening process was performed on LB agar plates supplemented with kanamycin. This was followed by a colony PCR and sequencing procedure, which served to confirm the integration of the gene. Beneficial mutants were identified by comparing yield changes and analyzing the results of the DNA sequencing.

### 2.3 Recombinant vectors construction

For the TB24 strain, primers dldh-F and dldh-R were used to amplify the dldh* gene from the optimal mutant, while gldh-F and gldh-R primers were utilized for amplifying the *gldh* gene from pET28a-*gldh*. The polymerase chain reaction (PCR) method was used to linearize the pET-28a (+) vector. Primers pET28a-F and pET28a-R were employed to amplify pET-28a (+) in order to obtain a linearized pET-28a (+). The primer sequences are presented in Supplementary Table S1. The PCR conditions followed the temperature profile previously described. In order to optimize the expression levels of both gene fragments, a computationally designed RBS library was used, and the optimized RBS sequences were incorporated into the primers (Supplementary Table S2). Furthermore, the expression balance of the dldh* and *gldh* genes was fine-tuned to enhance production controlled by these exogenous genes, which involved incorporating the optimized RBS sequences into the primers.

The ligation of the dldh* and *gldh* genes was achieved through OE-PCR. Subsequently, the PCR ligation product was ligated into the linearized pET-28a (+) vector using a seamless cloning technique, resulting in the recombinant co-expression plasmid pET28a-dldh*-*gldh.*

### 2.4 High throughput screening based on droplet microfluidics

A schematic diagram of the encapsulation, culture and cell sorting of a single droplet is shown in Figure 1B. The core components of the microfluidic system are two chips, as depicted in Supplementary Figures S1, S2, one for droplet generation and the other for droplet sorting (Pattanayak et al., 2021). These chips were manufactured using polydimethylsiloxane (PDMS) and a SU8-3000 negative photoresist molding technique, following standard lithography procedures. Prior to use, the microfluidic channels were treated with Aquapel™ to render the channel surfaces hydrophobic (Karamitros et al., 2020).

Three syringe pumps (LSP02-3B, Ditron-tech) and an outlet channel were connected to the chip using PEEK tubing. For the detection of droplet, a collimated light source of a specific wavelength (633 nm for D-PLA detection) was employed. As the re-injected droplet passes through the detection channel, changes in the optical signal are transmitted to the detection system via a fiber optic and recorded by a data acquisition card connected to a computer. A detailed description of the fiber-optic SERS probe preparation and data acquisition system is given in the Supplementary Material. Transformed cells were cultured in LB medium at 37°C for 12 h. Subsequently, 1 mL of the culture solution was centrifuged at 6,000 rpm for 1 min, after which the cells were washed and resuspended in an equal volume of PBS buffer. The organisms were then diluted in PBS buffer containing 10 g/L PPA and 0.5 mM IPTG in order to achieve an optical density (OD_600_) between 0.05 and 0.1 for droplet preparation. The droplet preparation process is schematically illustrated in Figure 1B. The aqueous phase consisted of a suitably diluted suspension in PBS liquid medium, and the carrier oil phase was a fluorinated solution of HFE-7500 (3M) containing 2.0% (v/v) Pico surfactant (5% in HFE-7500, Sphere Fluids). The mixture was divided into four portions with flow rates of 10 μL/min and 4 μL/min, respectively. Subsequently, the mixture was injected into a microfluidic droplet fabrication chip to create bacterial suspensions and spacer oils, producing uniform droplets with a diameter of 30 μm. By meticulously controlling the flow rates of the aqueous and oil phases during the droplet generation phase, it ensured that each droplet, at most, contains a single *E. coli* cell. This encapsulation efficiency was verified through microscopic observation, conducted concurrently with the preparation process. The resulting cell-containing droplets were collected into 250 mL shake flasks and incubated for 12 h at 37°C in a vertical incubator without stirring. The droplet sorting procedure is illustrated in Figure 1B. The droplets were collected at a flow rate of 0.5 μL/min. The separated droplets were then separated with 20 μL/min of spacer oil (HFE-7500, 3M). The separated droplets were then detected using Raman spectroscopy at 633 nm. Droplets exhibiting a strong Raman signal were directed into the sorting channel for collection using electrical deflection, while those with a weaker signal were directed into the waste channel. It was assumed that D-PLA is relatively uniformly distributed throughout the cell and its surroundings, which would result in a representative Raman spectroscopic measurement of a single droplet. Consequently, the SERS spectra would show an apparent linear response to the concentration of D-PLA in a single droplet. To demonstrate this, different concentrations of D-PLA droplets were examined to identify their Raman relative intensities under the same conditions (from 0.1 to 10.0 g/L). Subsequently, an adequate quantity of 1H,1H,2H,2H perfluoro-1-octanol (Sigma Aldrich) was added to dissolve the collected droplets. kanamycin was applied to LB agar plates at a concentration of 50 μg/mL (Yuan et al., 2022). The plates were incubated at 37°C for 12 h. Following the initial screening using RH-RMSS, the positive mutants were subjected to further culturing for activity and thermal stability assessment, as well as whole-cell-catalyzed reactions in 96-well plates. The concentration of D-PLA was determined using high-performance liquid chromatography (HPLC). The clones obtained in 96-well and 24-well plates will be subjected to further whole-cell catalyzed screening in flasks with the objective of identifying the highest yielding strains, as depicted in Figure 1C. The specific whole-cell-catalyzed conditions for 24-well plates and shake flasks are outlined in the section entitled “Optimization of co-expression enzyme conditions”.

### 2.5 Molecular modeling and docking

The amino acid sequence of DLDH from fermenting *Lactobacillus* species was initially retrieved from the NCBI database. Subsequently, a reliable template with a corresponding PDB ID was identified using Protein BLAST (BLASTP) against the Protein Data Bank (PDB) for the construction of its model. A 3D homology model of DLDH was then constructed using the SWISS-MODEL online software (https://swissmodel.expasy.org/). Moreover, the 3D structures of PPA were generated using PubChem software. Molecular docking between the native DLDH and PPA was conducted using the Autodock 4.2 program, as depicted in Figure 1A. In accordance with the gene sequencing outcomes, base substitutions were introduced within the PyMOL program, and the mutated DLDH was subsequently subjected to molecular docking with PPA using the same methodology. The results of the molecular docking were visualized and analyzed using PyMOL, as depicted in Figure 3.

### 2.6 Optimization of co-expression enzyme conditions

The dldh* gene from the most promising mutant screened was ligated to the *gldh* gene and inserted into pET28a to obtain the recombinant plasmid pdg00. This plasmid was then introduced into the MG1655 host, resulting in the generation of the strain MG1655-pdg00. The strain MG1655-pdg00 was cultured overnight on LB solid medium supplemented with 50 mg/L kanamycin, and single colonies were selected for further analysis. Subsequently, the plasmid was then isolated for preliminary validation using agarose gel electrophoresis and plasmid PCR. Following the initial validation, the sample was submitted to Tsingke Biotechnology Co., Ltd. for sequencing to confirm the successful transformation. A single colony was selected and incubated at 37°C with agitation at 220 rpm overnight to prepare the seed solution. Subsequently, the seed solution was inoculated into 50 mL of fresh LB medium at an inoculum volume of 1%. Once the OD_600_ reached 0.6–0.8, an investigation was conducted into the effects of different conditions on the production of D-PLA. This included the utilization of varying concentrations of IPTG (0, 0.2, 0.4, 0.6, 0.8, 1.0 mM), different temperatures (15°C, 20°C, 25°C, 30°C, 35°C, 40°C), and diverse induction times. The bacteria were harvested by centrifugation at 6,000 rpm and 4°C for 10 min. The catalytic system comprised 50 mL with a substrate concentration of 20.0 g/L. The reaction temperature was maintained at 35°C, and the pH of the reaction system was controlled at 7.0.

### 2.7 Enzyme activity assay

The cell pellet from *E. coli* expressing pET28a-dldh* was resuspended in buffer A (20 mM potassium phosphate, pH 8.0, 500 mM NaCl, 20 mM imidazole) and disrupted by sonication on ice. The lysate was clarified by centrifugation, and the supernatant was loaded onto a Ni^2+^-nitrilotriacetic acid column pre-equilibrated with buffer A. The column was washed with buffer A, followed by elution with buffer B (500 mM imidazole). The target protein was subsequently collected and dialyzed against 20 mM potassium phosphate buffer (pH 8.0) in order to remove imidazole. All purification steps were conducted at 4°C. The reducing activity of DLDH on PPA was assessed using spectrophotometric methods, measuring absorbance at 340 nm, and the amount of NADH reduced was quantified. The enzyme reaction was conducted in a 250 mL shake flask containing a reaction system with 50 mL of 1 mM NADH, 10 mM sodium PPA, and 100 mM sodium phosphate buffer at pH 8.0, along with 50.0 mg/mL of appropriately diluted enzyme solution. Enzyme activity was defined as one unit when it could oxidize 1 μM NADH per min (Cheng et al., 2022). The kinetic parameters of the mutant *dldh* were calculated from the initial velocity double reciprocal plot. The initial reaction velocity was determined by maintaining a constant concentration of NADH at 0.5 mM while varying the substrate (PPA) concentration from 1–10 mM. The kinetic parameters including the Michaelis-Menton constant (K_m_), maximum reaction rate (V_max_), turnover number (K_cat_), and catalytic efficiency of PPA (V_max_/K_m_), were calculated using Lineweaver-Burk plots. All reactions exhibited Michaelis-Menten kinetics (Luo et al., 2020).

### 2.8 Production of D-PLA by expanding culture in bioreactor

The fermentation was performed in a 5 L bioreactor. Initially, 2.5 L of LB liquid medium was introduced into the bioreactor, followed by the addition of a 5% inoculum. Once the OD_600_ reached 0.6–0.8, the cells were induced with IPTG to a final concentration of 0.2 mM under the following conditions: The temperature was maintained at 25°C, the agitation rate was set at 220 rpm, and the incubation period was 15 h. Subsequently, the cell cultures were concentrated and resuspended in 1 L of PBS buffer (pH 7.0) containing 80.0 g/L of polyphenol-rich extract (PPA) and 30.0 g/L of glycerol. The biotransformation was conducted in a 5 L bioreactor at 220 rpm, pH 7.0, and 35°C.

### 2.9 Analytical method

The substrate PPA and the products D-PLA and DHA were quantified using high-performance liquid chromatography (HPLC). For sample preparation, whole-cell catalytic samples were centrifuged at 12,000 rpm for 5 min, and the supernatant was filtered through a 0.22 μm sterile organic filtration membrane. Subsequently, the filtrate was diluted 100-fold with PBS buffer at a pH of 7.0. For the determination of D-PLA concentration, an Agilent ZORBAX Eclipse XDB C18 chromatographic column (5 μm, 4.6 mm × 250 mm) was used, with a mobile phase consisting of an organic phase (0.05% trifluoroacetic acid in methanol) and an aqueous phase (0.05% trifluoroacetic acid in water). Detection was conducted at a wavelength of 210 nm, with a flow rate of 1.0 mL/min. The temperature of the column was maintained at 20°C, and an injection volume of 10 µL was used. A mobile phase ratio of 50:50 (v/v) was employed for this analysis. The D-PLA standard showed good linearity in the concentration range of 0.04–0.64 g/L, as shown in Supplementary Figure S7.

Similarly, specific conditions were established for the detection of DHA. A Shim-Nex CS C18 column (5 μm, 4.6 mm × 250 mm) was utilized. The mobile phase consisted of an organic phase (methanol solution) and an aqueous phase (0.05% aqueous phosphoric acid solution). The detection of DHA was conducted at a wavelength of 272 nm, with a flow rate of 1.0 mL/min. The column temperature was maintained at 25°C, and an injection volume of 10 µL was employed. The time program for this analysis involved a mobile phase ratio of A:B = 5:95 (v/v). The DHA standards showed good linearity in the concentration range of 0.5–10 g/L as shown in Supplementary Figure S8.

## 3 Results and discussion

### 3.1 Droplet microfluidic mediated optimal mutant screening

**FIGURE 2:**
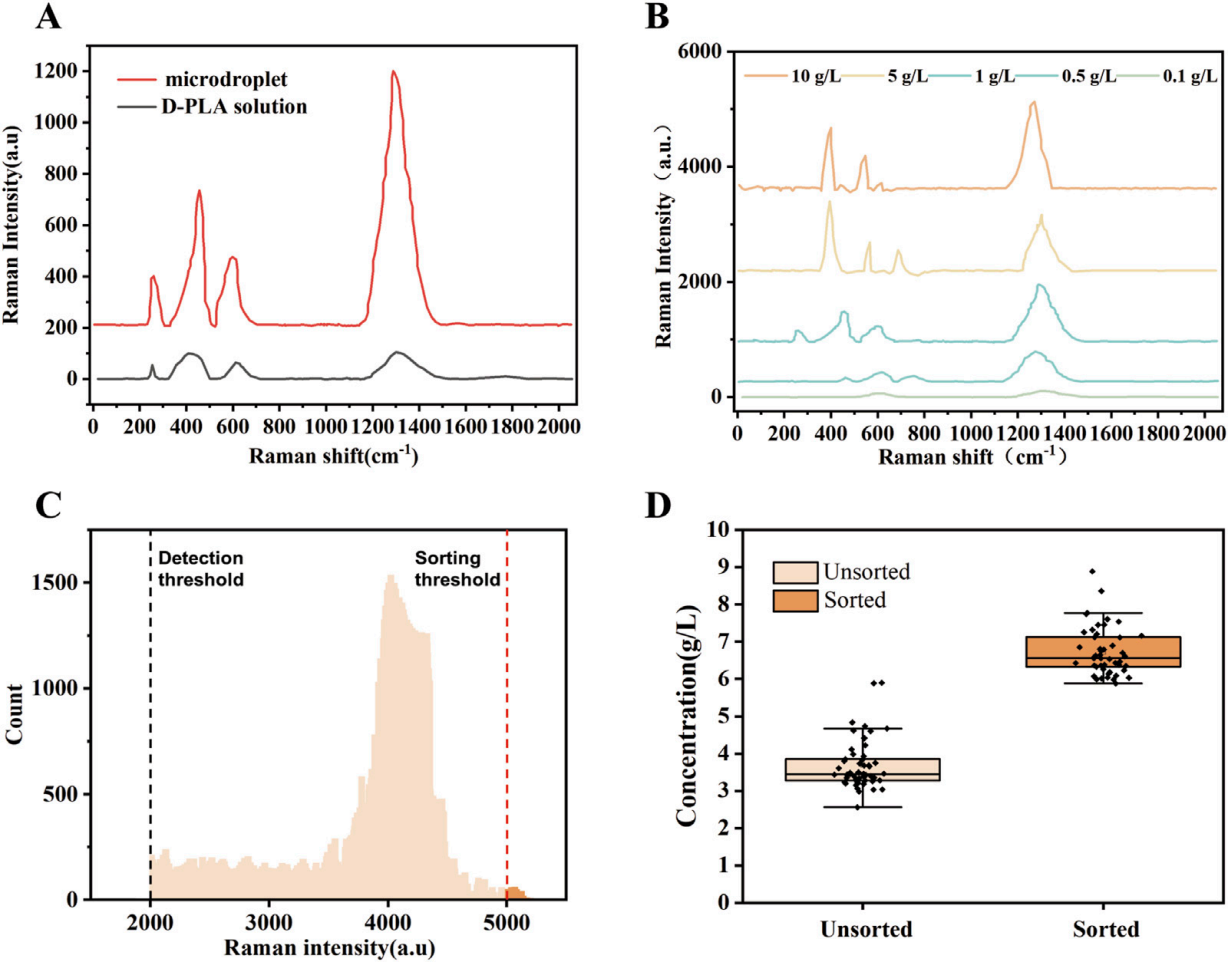
Design and validation of quantitative Raman detection of D-PLA levels. **(A)** Comparison of Raman signals among D-PLA solutions and simulated droplets, with the main Raman bands positioned at 1,285 cm^−1^ **(B)** SERS spectra of D-PLA droplets with different concentrations. **(C)** A demonstration of significant enrichment from an artificially mixed droplet library, with boxplots showing D-PLA concentrations from different sorted populations. Before sorting, n = 50 independent colonies were selected from the designed mixed library; after sorting, droplets were selected based on Raman intensities, with n = 50 independent colonies from the sorted pool. **(D)** A histogram showing the distribution of droplet Raman intensities for the sorting of the *dldh* random mutagenesis library. Droplets that surpassed the sorting threshold (indicated by a red dashed line) were sorted and collected.

Random mutagenesis is a widely used technique, often employed in conjunction with screening or selection processes to identify mutant enzymes with desired properties (Cravens et al., 2021). D-LDH is the rate-limiting enzyme in the biosynthesis of D-PLA. Therefore, in this study, an error-prone PCR-based D-LDH mutant library was created to improve the catalytic activity. Approximately 10^9^ mutant libraries were generated using the above PCR system. As depicted in Figure 1B, single *E. coli* cells containing random *dldh* mutants were encapsulated in droplets, incubated, induced, and fermented. The fermented droplets were then re-injected into the droplet sorting device for HTS. The droplet sorting procedure adhered to the standard protocol, which is described in the Materials and Methods section. The system utilized the intensities of the characteristic Raman peaks of D-PLA and the higher the relative intensity of these characteristic peaks, the higher the concentration of D-PLA in the droplet. To confirm the presence of D-PLA in a microdroplet, a standard solution of D-PLA was compared with the signal from a microdroplet simulating a mature cell. The spectra obtained showed identical profiles, indicating that D-PLA was successfully detected in the microdroplets, as depicted in Figure 2A. Notably, the Raman spectra showed a linear response, indicating an approximate analyte concentration. Encouragingly, the average relative Raman intensities near 1,276 cm^−1^ in the range of 0.1–10.0 g/L showed excellent correlation, and therefore, the relative Raman intensities in this range were used as a benchmark for screening, as depicted in Figure 2B. The SERS intensity in the range of 1,276 cm^−1^ gradually with increasing D-PLA concentration, demonstrating a strong linear relationship with D-PLA concentration (R^2^ = 0.998), as depicted in Figure 1D. These results validate the potential of SERS as a tool for quantitative analysis of D-PLA within single cells. Based on these results, the relative Raman intensity at 1,250–1,350 cm^−1^ was selected as the detection target for subsequent sorting experiments.

After droplet sorting 3 h, about 2 million droplets were screened and sorted, identifying mutant strains produced 1.4 times or more D-PLA than the CP303 strain in these droplets. The statistical droplet histogram in Figure 2C showed the distribution of the droplet signal. The detection limit of SERS for D-PLA could reach 1 × 10^−8^ g/L, demonstrating the high sensitivity of SERS for the detection of D-PLA in the droplets. The sorted strains were extracted from the collected droplets, plated on LB agar, and cultured for 12 h. Individual colonies were isolated and inoculated into 96-well plates for further screening. Ten strains with high relative Raman intensities were obtained. These strains were subsequently subjected to whole-cell catalytic validation under the conditions described in the “Optimization of co-expression enzyme conditions” section. All of these strains showed excellent D-PLA production capabilities, with the top performer reaching a D-PLA production level of 8.32 g/L, as shown in Supplementary Figure S3. This represents a 52.66% increase compared to the original strain, designated TB24.

The results demonstrated that the high-throughput method developed in this study, which utilizes the SERS signals to indicate the concentration of D-PLA, represents a promising option. The high-throughput droplet microfluidic sorting system developed has proven to be highly efficient and effective for determining D-PLA concentration. To validate the sorting efficiency, an artificially mixed droplet library was prepared by mixing droplets containing *E. coli* DH5α. After cultivation and fermentation, these mixed droplets were sorted by RH-RMSS with the sorting criterion set for Raman shifts between 1,250 and 1,350 cm^−1^ to be greater than 4,000. 50 of the sorted droplets were randomly selected and cultured in a 24-well plate for fermentation. They were then compared with 50 unsorted strains using HPLC to determine the D-PLA concentration. The results demonstrate a significant increase in D-PLA production in the 50 sorted mutant cells compared to the unsorted cells, as depicted in Figure 2D.

Various studies have demonstrated the feasibility of applying Raman spectroscopy to HTS. A combined multivariate analysis method has been developed using a porous silicon SERS substrate, which can accurately analyze and identify Phe and carotenoids (Yue et al., 2020). Additionally, a technique using a fiber-optic switch in combination with a SERS probe has been developed, capable of analyzing multiple (n = 8) samples rapidly and with good sensitivity, applicable to proteins, bacteria, and more. A qualitative and quantitative detection method was established for aristolochic acid using a simple SERS method, which showed good linearity in the range of 0.2–120 M. The results showed that the quantitative and qualitative detection of aristolochic acid is highly sensitive (Meng et al., 2023). This indicated that the SERS-based microdroplet screening method developed in this study is undoubtedly effective for the HTS of D-PLA.

### 3.2 Enzyme activity and molecular docking termination analysis of selected variants

Enzyme activity is an indicator of the quality of enzyme mutants. Therefore, the enzyme activity of the mutants was measured to verify the cause of increased D-PLA production. The apparent kinetic parameters K_m_ and K_cat_ of *dldh* gene in both the mutant and original strains were determined on PPA to compare the enzyme expression levels, as depicted in Table 1. The results showed that the reaction pattern fitted well with the Michaelis-Menten equation. The K_m_ value of *dldh* ^A174Q\V331R^ showed a 72.6% decrease and a 5.31-fold increase in K_cat_ value compared to the K_m_ and K_cat_ values of the original *dldh* (4.38 mM and 51.20 s^−1^). Thus, the catalytic efficiency (K_cat_/K_m_) of *dldh* ^A174Q\V331R^ (269.27 mM^−1^ s^−1^) was 23.03-fold higher than that of the original *dldh*, as depicted in Figure 1E. The kinetics of *dldh* before and after mutation were analyzed using different concentrations of PPA. As shown in Table 1, the kinetic parameters were determined using Lineweaver-Burk plots and the results were compared with data from a previous study (Lee et al., 2019). The K_cat_/K_m_ value of *dldh* against PPA before mutation was 11.69, while the highest K_cat_/K_m_ value after mutation was found in the microtiter-screened mutant (269.27). This result indicates that the mutant *dldh* has an efficient reducing activity against PPA (K_m_ = 1.20 mM, K_cat_ = 323.12 s^−1^). These changes in the kinetic parameters of the enzyme suggest that the substitution of residues A174Q\V331R has a positive and synergistic effect on the substrate acceptability of *dldh* and its affinity for PPA. This is similar to the results of a previous study that used targeted mutagenesis of key residues to increase DLDH enzyme activity to 1336.39 mM/s (Cheng et al., 2022).

**TABLE 1:** Kinetic parameters of DLDH (*dldh*) and its mutant form (dldh*) were determined using PPA as the substrate. All reactions were performed in triplicate.

Strains	Enzymes	V_max_ (μM s^−1^)	K_m_ (μM)	K_cat_ (s^−1^)	K_cat_/K_m_ (s^−1^ μM^−1^)
CP303	dldh	224.26 ± 2.95	4.38 ± 0.78	51.20 ± 2.3	11.69
TB00	dldh*	387.74 ± 3.34	1.20 ± 0.15	323.12 ± 3.8	269.27

**FIGURE 3:**
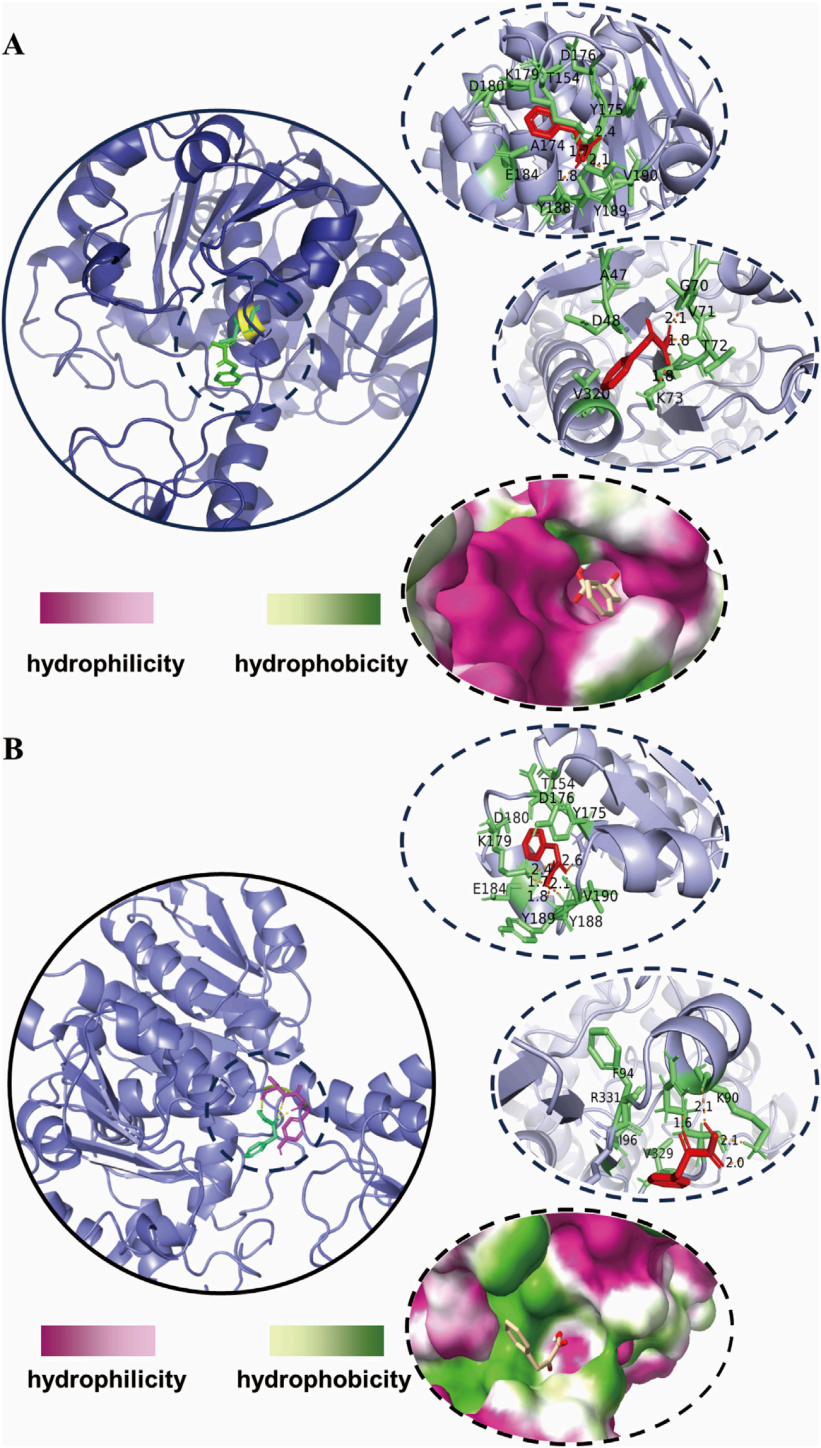
Molecular docking simulations of enzyme-substrate binding **(A)** simulation of the docking of the wild-type DLDH with PPA **(B)** simulation of the docking of the mutant-type DLDH with PPA. The shade of color in the binding pockets indicates the degree of hydrophobicity.

Ten robust strains with elevated D-PLA production capabilities were successfully identified through a meticulous screening process. Initially, isolated droplets were incubated, and individual colonies were selected for inoculation into 96-well plates, facilitating subsequent rounds of screening. To elucidate the genetic basis for the enhanced D-PLA yield observed in these strains, a detailed analysis of mutations within the *dldh* gene was conducted through DNA sequencing. The sequencing outcomes, which reveal the underlying genetic variations, are presented in Table 2. Upon comparative analysis with the wild-type sequences of *dldh*, it was observed that all strains, with the exception of strains 5 and 7, exhibited mutations at the A174Q and V331R loci. These findings have prompted us to hypothesize that the mutations at A174Q and V331R are the primary contributors to the enhanced D-PLA production capacity observed in these particular strains. Intriguingly, strains 5 and 7 also displayed mutations in proximity to site 174. To further unravel the intricacies of the enzyme activity increase, a comprehensive investigation of these mutations was undertaken. Computer simulation methods have been demonstrated to be highly effective in predicting enzyme stabilization or activity enhancement (Marques et al., 2021; Kroll et al., 2023). Amino acid residues situated at the core of enzyme activity, including those within the catalytic pocket and tunnel, have been demonstrated to significantly influence the catalytic activity of the enzyme (Ribeiro et al., 2020). To further elucidate the mechanism of the enhanced catalytic activity of the mutant, the crystal structure of *Lactobacillus* fermentum LDH (PDB ID: 2DLD) was used as a template to simulate the structures of both the wild type (WT) and mutant TB24. The substrate PPA was found to dock with the enzyme’s active center, inducing a conformational change in the active site upon close proximity. The substrate primarily binds to the enzyme through hydrophobic interaction, initiating the catalytic reaction. It can therefore be concluded that the hydrophobic binding between the active center and the substrate, as well as the conformation of the active center, significantly impacts the activity of the enzyme. The binding of the parental wild type to the substrate sodium PPA is illustrated in Figure 3A, where the protein binds to the substrate via four hydrogen bonds, formed with tyrosine at position 188 and valine at position 190. Moreover, the hydrophobicity of the binding pocket of the ancestral enzyme was modeled, revealing that despite the substrate binding to the ancestral enzyme with a deeper pocket, it has a reduced number of hydrophobic amino acid residues within the binding pocket. In contrast, the simulation results for the mutant enzyme-substrate binding are presented in Figure 3B. When the amino acid at position 174 changes, the protein-substrate attachment relies on five different hydrogen bonds, involving tyrosine at position 189, valine at position 190, and lysine at position 179. When the amino acid at position 331 is changed, the number of hydrogen bonds binding the substrate to its residues increases from three to four. Additionally, the simulation of the hydrophobicity of the mutant enzyme’s binding pocket demonstrated that hydrophobic amino acid residues surrounding the binding pocket are more prevalent. Consequently, the enhanced catalytic activity of the mutant enzyme could be attributed to the expansion of the binding site and the augmented hydrophobicity of the binding pocket.

**TABLE 2:** Mutation results for the ten strains with the highest D-PLA yield.

Strain	Mutation site
1	K90R/A174Q/V331R
2	K34E/A174Q
3	K34E/A174Q/V331R
4	A174Q/V331R
5	K34E/F178I/I286T
6	A174Q/V331R
7	F178I/I286T
8	A174Q/I286T/V331R
9	K34E/A174Q/V331R
10	A174Q/V257A/V331R

These results are comparable to those of recent studies, such as the replacement of Phe and alanine with tryptophan and tyrosine, respectively, which enhanced the hydrophobic interactions between the enzyme and the substrate, thereby improving the catalytic efficiency of DLDH (Zhou Y. et al., 2024). The dldh* denotes a variant with mutations at the A174Q and V331R loci. The enhanced activity of the dldh* enzyme can be primarily attributed to two key factors: an augmentation in hydrogen bonding between the substrate and the enzyme, and an increase in the hydrophobicity of the substrate binding pocket. The elevated enzymatic activity of the mutant enzyme, dldh*, significantly contributes to the increased production of D-PLA.

### 3.3 Replacement of chassis cells to increase D-PLA production

In the aforementioned studies, the enzyme activity of DLDH, a pivotal enzyme in the synthesis of D-PLA, has been enhanced through directed evolution and HTS. However, during the whole-cell-catalyzed synthesis of D-PLA in *E. coli* BL21, a low abundance of soluble DLDH proteins was observed, with the majority forming inclusion bodies, which limited D-PLA production. Therefore, given the ease of industrial-scale cultivation of *E. coli*, we opted for *E. coli* MG1655 as the chassis cell for further investigations (Shi et al., 2024). It has been demonstrated that *E. coli* MG1655 is less susceptible to inclusion body formation and exhibits a higher growth rate compared to BL21 for protein expression (Wang et al., 2020). To address this challenge, research has focused on optimizing protein expression conditions, aiming to minimize inclusion body formation and thereby increase the soluble expression of target proteins.

**FIGURE 4:**
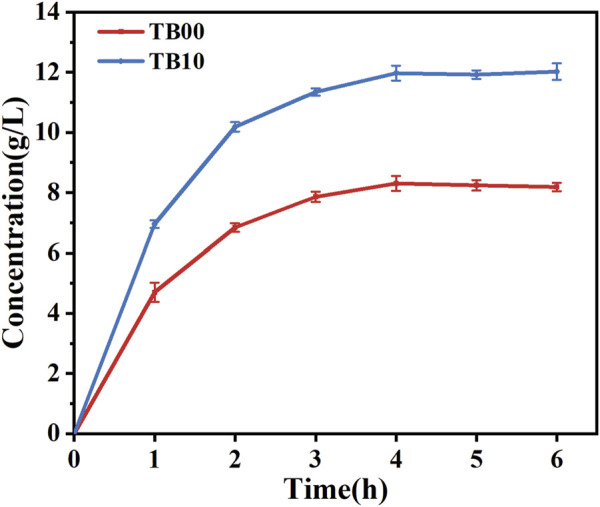
Comparison of D-PLA yields before and after chassis cell replacement. TB00 is an *Escherichia coli* BL21 (DE3) strain containing the plasmid pdg00, while TB10 is an *E. coli* MG1655 (DE3) strain also containing the plasmid pdg00. The catalytic system consisted of a 50 mL PBS solution containing PPA at a concentration of 20.0 g/L and glycerol at a concentration of 15.0 g/L. The reaction temperature was maintained at 37°C, and the pH of the reaction system was controlled at 7.5. All reactions were performed in triplicate, with error bars representing the standard error of the mean.

The plasmid pdg00 (pET28a harboring the genes dldh* and *gldh*) was introduced into the MG1655 host to obtain the strain MG1655-pdg00, which was designated as TB10. The catalytic reaction was conducted in 50 mL of PBS buffer (pH = 7.0) by incubating and inducing TB10 with TB00 under identical conditions to ascertain the catalytic efficiency of strain TB10. Strain TB00 is an *E. coli* BL21 (DE3) strain harboring the pET28a plasmid, expressing a double mutant DLDH^A174Q/V331R^ and GLDH. The results demonstrated that, under identical conditions, the catalytic efficiency of TB10 was significantly higher than that of TB00, with TB10 exhibiting an approximate 45.43% increase in D-PLA yield after 6 h, as depicted in Figure 4. It is hypothesized that the higher soluble protein levels in TB10 may lead to a corresponding increase in D-PLA yield. To further elucidate the reasons behind the observed increase in D-PLA yields in TB10 relative to the TB00 strain, a comparative analysis was conducted. This analysis assessed the growth dynamics of both strains under identical conditions. OD_600_ revealed that the MG1655 strain, harboring the pTg00 plasmid, exhibited more robust growth compared to BL21 (DE3), as depicted in Supplementary Figure S4. This finding supported our hypothesis that inclusion body formation can inhibit cellular growth. Furthermore, it was confirmed that the MG1655 strain is less prone to forming inclusion bodies, thereby conferring a growth advantage over the BL21 (DE3) strain, consistent with its reported higher stress tolerance. To substantiate this hypothesis, SDS-PAGE analysis was conducted, and the results demonstrated a significant enhancement in the soluble protein expression of both DLDH and GLyDH in TB10, as shown in Supplementary Figure S5.

These results are comparable to previous studies where a commercial molecular chaperone for pG-KJE8 was employed to enhance the solubility of β-carotene ketolase, resulting in a 30.77-fold increase in carotenoid production (Zhou Q. et al., 2024). The findings indicated that the co-expression of chaperone proteins is a viable strategy for enhancing the folding of target proteins and preventing the formation of inclusion bodies. Although this study experimented with the chassis cell replacement strategy and achieved positive outcomes, it is undeniable that the co-expression of chaperone proteins represents an exemplary strategy in its own right. Consequently, future studies may focus on exploring this approach in greater depth, with the aim of maximizing its potential for optimizing protein production and folding. This phenomenon is primarily attributed to the overexpression and misfolding of target proteins, which results in the aggregation of proteins in an unfolded state within inclusion bodies. Investigations have been conducted to enhance the solubility of expressed proteins by regulating induction temperature and IPTG concentration. These adjustments have led to improved solubility of expressed proteins (Gutiérrez-González et al., 2019). Augmenting the soluble protein content has been demonstrated to have a significant impact on the yield of D-PLA. The enhancement of soluble protein levels is a critical factor in improving the production and accumulation of D-PLA, suggesting that it may serve as an effective strategy for optimizing the output of this biopolymer.

### 3.4 RBS sequence substitution and condition optimization

The directed evolution process increased the activity of the key enzyme for D-PLA synthesis. However, the synthesis of D-PLA requires a significant amount of cofactor NADH. Consequently, numerous studies have been conducted with the objective of regenerating cofactors for D-PLA synthesis. The SDS-PAGE results from the fraction of replacement chassis cells that increased D-PLA production showed that the expression of GLyDH was suboptimal, leading to a deficiency of NADH for the D-PLA synthesis process. As a result, D-PLA production was negatively affected. The optimization of RBS sequences can significantly adjust gene expression. Furthermore, the rational up-regulation of key enzymes and the down-regulation of non-key enzymes can further increase the yield of target metabolites (Rao et al., 2024).

**FIGURE 5:**
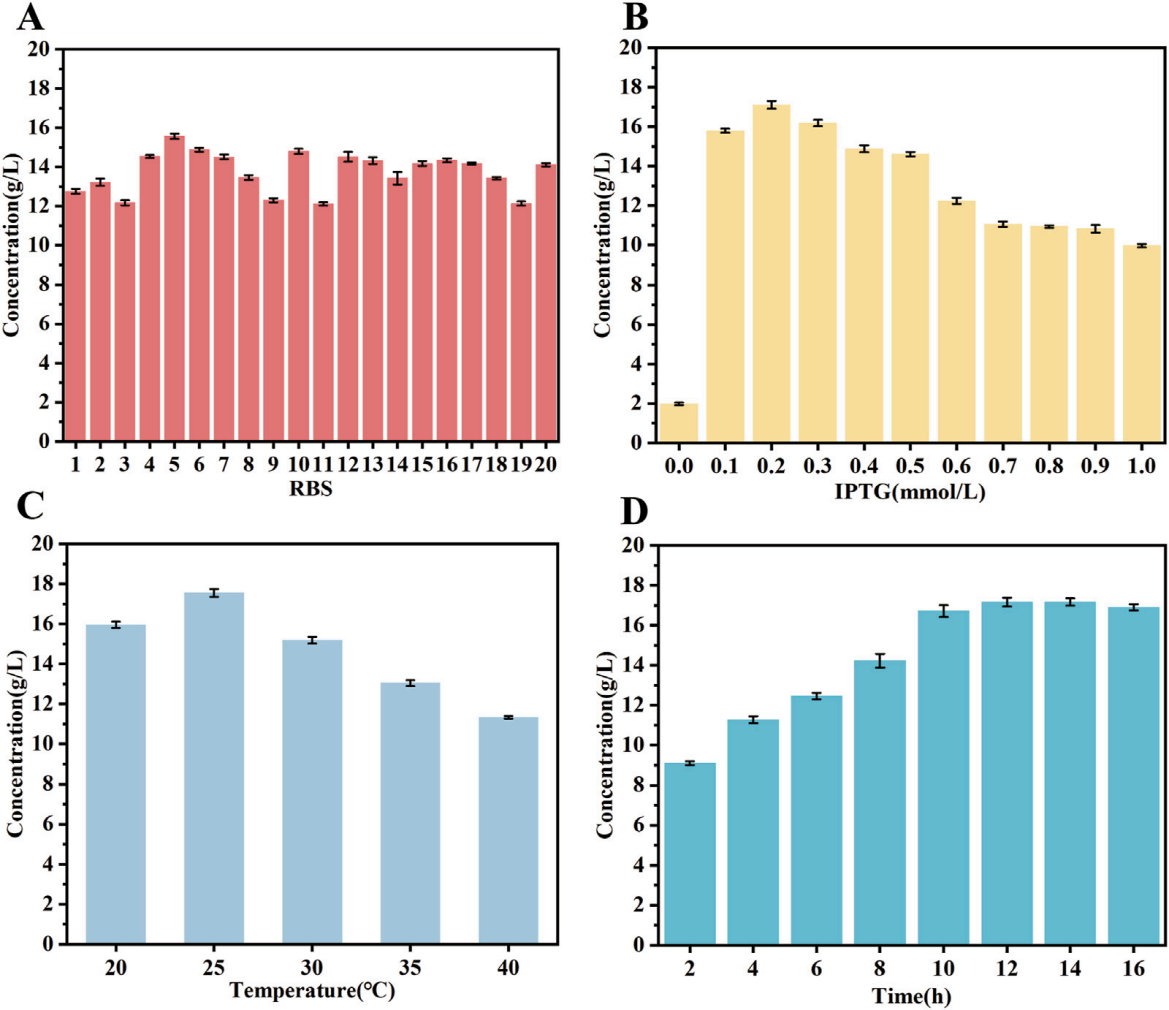
Exploration of optimal expression conditions for the mutant enzyme. **(A)** RBS sequences optimization **(B)** IPTG concentration **(C)** induction temperature **(D)** induction time. The catalytic system comprised 50 mL of a solution containing PPA substrate at a concentration of 20 g/L and glycerol at a concentration of 15 g/L. The reaction temperature was maintained at 37°C, the pH of the reaction system was controlled at 7.5, and the reaction time was 6 h. All reactions were performed in triplicate, with error bars representing the standard error of the mean.

Therefore, 20 RBS sequences with higher intensities were selected from the Anderson RBS library with the expectation of coordinating the expression of DLDH and GLyDH and further increasing the yield of D-PLA. The findings indicate that the majority of the RBS sequences effectively enhanced the production of D-PLA, with sequence R5 exhibiting the most promising performance. In particular, R5 significantly increased D-PLA yield from 12.1 to 15.67 g/L, as depicted in Figure 5A. This augmentation suggests that the expression of GLyDH may have been upregulated, thereby providing an adequate supply of the cofactor NADH for production of D-PLA. The observed increase in activity indicates a potential upregulation of GLyDH expression, which facilitates the production of sufficient cofactor NADH for the biosynthesis of D-PLA. Further examination of the samples via mass spectrometry analysis revealed the presence of dihydroxyacetone, which is hypothesized to be an oxidation product of glycerol. This finding implies that additional metabolic intermediates may be involved in the overall production process.

The objective of this study was to investigate the impact of inducer (IPTG) concentration, induction temperature and induction time on protein expression in strain TB15. Strain TB15 is an *E. coli* BL21 (DE3) harboring pET28a, expressing a double mutant DLDH^A174Q/V331R^ and GLDH, with DLDH linked to GLDH via the R5 RBS sequence. The results demonstrated that the production of D-PLA was optimized when 0.2 mM IPTG was added to the medium, as depicted in Figure 5B. Furthermore, the data indicated that the optimal yield of D-PLA was achieved when gene expression was induced at temperatures ranging from 15°C to 40°C, with the highest yield observed at an induction temperature of 25°C (Figure 5C). Finally, the effect of induction time on protein expression was further investigated at an IPTG concentration of 0.2 mM and an induction temperature of 25°C. The results demonstrated that the production of D-PLA peaked at an induction time of 15 h, as depicted in Figure 5D.

Researchers have optimized the production of 4-HIL by selecting RBS sequences with varying strengths, with the highest strength RBS sequence increasing the yield of 4-HIL by approximately 9.9% (Shi et al., 2020). The expression levels of GLyDH and DLDH before and after RBS sequences optimization were analyzed using SDS-PAGE, as depicted in Supplementary Figure S6. The expression of GLyDH was significantly enhanced after replacing the RBS sequence with R5, which could potentially provide a more abundant supply of the cofactor NADH for D-PLA production under identical condition. Moreover, the replacement of this RBS sequence did not significantly affect the expression of DLDH, thus effectively enhancing D-PLA production. The expression of soluble proteins in *E. coli* is influenced by a number of factors, including the choice of vector, cloning method and bacterial growth conditions (Gutiérrez-González et al., 2019). The type of promoter used is critical because it affects the level of protein expression by influencing the amount of protein produced after induction. Therefore, changing promoters has become a strategy to improve protein production. A method known as “ReToAd” for rapid promoter replacement in the laboratory has been demonstrated to enhance protein yields by evaluating the effects of various promoters, such as A3, Lac, Pac, SP6, Tac, Trc, and T7. The results showed that the activity of the GDH enzyme, when expressed in *E. coli* with the Trc promoter, was 31 times higher than that expressed with the T7 promoter (Cheng et al., 2018). Further studies, which utilized a more robust constitutive promoter, revealed through transcriptional analysis that the conversion of riboflavin to flavin mononucleotide by the riboflavin kinase encoded by the FMN1 gene is the rate-limiting step in the synthesis of flavin adenine dinucleotide (FAD). Consequently, by replacing the native promoter of the FMN1 ORF with the more potent constitutively expressed GPD (glyceraldehyde-3-phosphate dehydrogenase) promoter, riboflavin kinase activity increased 35.67-fold, and FAD production increased 14.02-fold (Patel and Chandra, 2020). Coincidentally, the same researchers further increased acrylic acid production by using the constitutive BBa_J23100 promoter to replace the native promoter for expressing the upstream gene involved in acrylic acid production (Ko et al., 2020). In light of these insights, it is plausible that the T7 promoter could potentially be outperformed by either constitutive promoters or inducible promoters that utilize cost-effective inducers. Given this, the exploration of this strategy in our forthcoming research is being inclined towards. Furthermore, the integration of target genes into the genome will be explored within our research endeavors, aiming to enhance efficiency.

**FIGURE 6:**
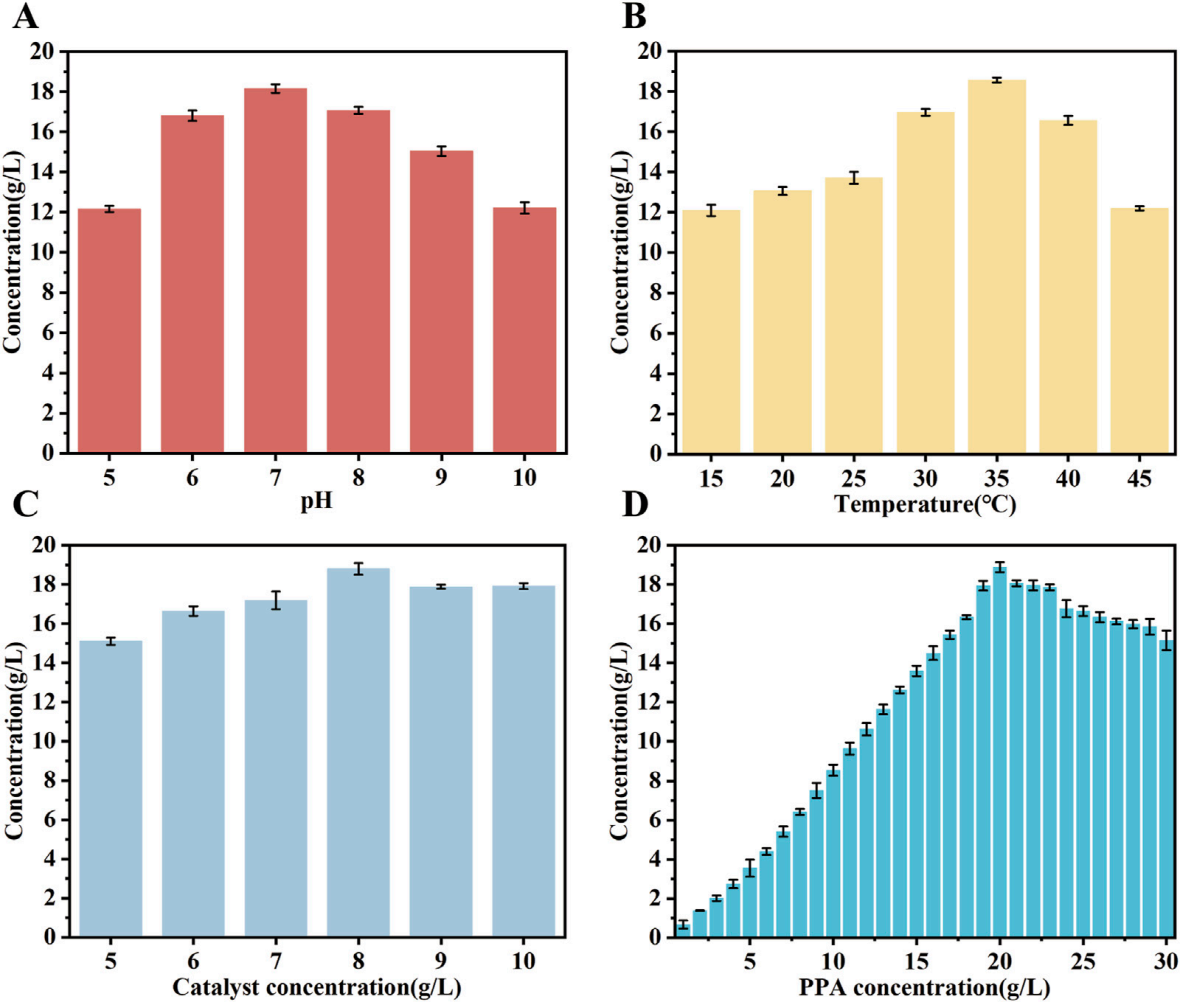
The performance of biocatalysts was evaluated under different conditions. **(A)** the effect of catalytic pH on D-PLA production **(B)** the effect of catalytic temperatures on D-PLA production **(C)** the effect of crude enzyme solution concentration on D-PLA production **(D)** the effect of substrate concentrations on D-PLA production. The induction conditions were standardized to 15 h at 25°C with a 0.2 mM inducer concentration. The crude enzyme solution was obtained by centrifugation at 6,000 rpm and 4°C for 10 min. All reactions were performed in triplicate, with error bars representing the standard error of the mean.

Studies have demonstrated that the optimal temperature for the expression of the mutase enzyme can vary significantly (Zhang et al., 2022). In order to enhance the expression of the mutase biocatalyst, its performance was conducted under a variety of conditions. The experiments employed a PPA concentration of 20 g/L. The results indicated that the catalyst exhibited peak activity at a pH of 7.0, maintaining activity levels above 80% between pH 7.0 and 9.0, as depicted in Figure 6A. Moreover, the biocatalysts exhibited an optimal temperature range of 20°C–45°C, with the highest catalytic efficiency observed at 35°C. Activity declined as the temperature increased from 35°C to 45°C, as depicted in Figure 6B. The optimal reaction rate was found at a concentration of 8 mg/mL of the crude enzyme solution, as depicted in Figure 6C. Subsequently, the impact of substrate concentration on the reaction rate of the mutant strain was evaluated within the range of 1–30 g/L, as depicted in Figure 6D. These findings demonstrated that D-PLA production increased with higher PPA concentrations, peaking at 20 g/L and resulting in the generation of 18.86 g/L of D-PLA by strain TB15. Attempts to produce D-PLA at even higher substrate concentrations did not yield the desired outcome under identical experimental parameters. Conversely, elevated substrate concentrations resulted in the inhibition of DLDH enzyme activity, thereby reducing the reaction rate. The results demonstrated that the optimal expression conditions for the mutant enzyme differ from those of the wild-type. Moreover, the optimization of enzyme expression conditions has been demonstrated to markedly enhance the yield of D-PLA, underscoring the pivotal role of enzymatic condition refinement in improving productivity.

### 3.5 Fed-batch fermentation of D-PLA

**FIGURE 7:**
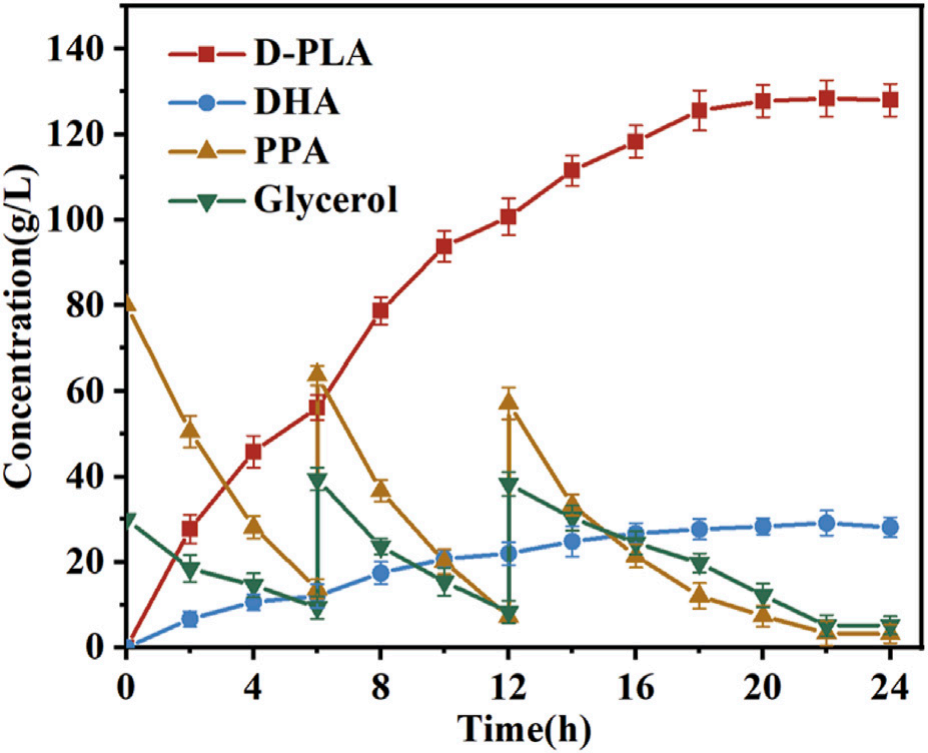
Assessment of D-PLA production was conducted in 5-L bioreactors. The reaction system, with a volume of 3 L, was maintained at 25°C, and consisted of a PBS buffer (pH = 7.0) containing 80 g/L of PPA and 30 g/L of glycerol. In addition, 8 g/L of whole-cell catalyst was added to the system. As the reaction progressed, the accumulation of D-PLA was observed concurrently with the consumption of PPA. All reactions were performed in triplicate, with error bars representing the standard error of the mean.

The bioconversion process in fermenters frequently entails the utilization of batch feeding techniques within bioreactors (Abadli et al., 2021). The impact of continuous feeding on the synthesis of D-PLA and DHA in bioreactor was evaluated, based on the optimal induction and incubation conditions identified in previous shake flask experiments. In particular, the reaction was a conversion process for the production of D-PLA in a 5 L bioreactor. When the concentration of PPA in the reaction system fell below 10.0 g/L, solid sodium PPA (50.0 g) was added. When the glycerol concentration in the reaction system dropped below 10.0 g/L, it was replenished to 20.0 g/L. Samples were collected at hourly intervals from the commencement of the reaction for subsequent HPLC analysis. As illustrated in Figure 7, the final conversion of PPA reached 88%, and the D-PLA yield reached up to 128.4 g/L. It is noteworthy that DHA, the oxidation product of glycerol, also exhibited a yield of 29.1 g/L. These results indicated that the production method described has potential for industrial application. The implementation of optimization strategies led to a notable enhancement in the D-PLA titer, yield, and productivity of the TB15 strain, exhibiting increases of 37.62%, 5.86%, and 10.17%, respectively, compared to the CP303 strain. This improvement demonstrated the efficacy of the optimization techniques in enhancing the performance of TB15 for D-PLA production. In the meantime, the results demonstrated the efficacy of the Raman-based microdroplet screening system developed in this study for strain screening. The results robustly confirmed the effectiveness of the Raman microdroplet screening system in strain screening, verifying its suitability.

## 4 Conclusion

In this study, error-prone PCR was employed to generate a diverse mutant library of DLDH. A mutant strain exhibiting significantly enhanced DLDH activity was successfully isolated by utilizing a SERS-based microtiter screening method. To facilitate the regeneration of the cofactor NADH during the synthesis of D-PLA, GLyDH and the optimized DLDH mutant were co-expressed in *E. coli* MG1655. Notably, the yield of D-PLA from the screened mutant strain exceeded that of the original strain by approximately 52%, which indicated the substantial benefits of the genetic engineering approach employed. The enhanced D-PLA yield of the dominant mutant strain was attributed to an increase in substrate-enzyme binding hydrogen bonding and an elevated hydrophobicity of the binding pocket, as evidenced by molecular docking simulations. The expression levels of the dual enzyme system were regulated by optimizing the RBS sequences to increase the supply of the cofactor NADH. The strategy employed resulted in shake flask yields of up to 18.84 g/L of D-PLA, representing a 3.2-fold increase in production compared to the initial catalytic results. Moreover, at the level of the 5 L bioreactor, the maximum yield of D-PLA reached to an advanced level of 128.4 g/L with the yield of DHA also being as high as 29.1 g/L. This work provides a reference point for the whole-cell-catalyzed production of D-PLA and offers insights into enzyme modification. It also serves as an example of a SERS-based microdroplet screening platform, demonstrating its potential for high-throughput screening of high-yielding strains for the production of high-value chemicals.

## Data Availability

The raw data supporting the conclusions of this article will be made available by the authors, without undue reservation.
