# Corrigendum: SERS-based microdroplet platform for high-throughput screening of *Escherichia coli* strains for the efficient biosynthesis of D-phenyllactic acid

**DOI:** 10.3389/fbioe.2024.1527255

**Published:** 2024-11-26

**Authors:** Lin Hu, Ruoshi Luo, Dan Wang, Fanzhen Lin, Kaixing Xiao, Yaqi Kang

**Affiliations:** Department of Chemical Engineering, School of Chemistry and Chemical Engineering, Chongqing University, Chongqing, China

**Keywords:** D-phenyllactic acid, surface-enhanced Raman spectroscopy, microdroplet screening, directed evolution, molecular docking

In the published article, there was an error in the **Funding** statement. The funding number, “(2022YFC2105700),” was incorrect: “This work was supported by the National Key Research and Development Program of China (2022YFC2105700); the National Natural Science Foundation of China (22378032); Chongqing Outstanding Youth Fund (cstc2021jcyj-jqX0013), the Human Resources and Social Security Bureau of Chongqing (cx2023036).” The correct Funding statement appears below.

