# Supplementary material for: SERS-based microdroplet platform for high-throughput screening of Escherichia coli strains for the efficient biosynthesis of D-phenyllactic acid
Source: Front Bioeng Biotechnol. 2024 Sep 20;12:1470830. doi: 10.3389/fbioe.2024.1470830 (PMC11449890; doi:10.3389/fbioe.2024.1470830)
Supplement: Supplementary file 1 [file Table1.DOCX]

**SERS-based high-throughput microdroplet screening for substantially improved D-PLA biosynthesis in *Escherichia coli*.**

The microdroplet screening method in this thesis mainly includes the following three steps: (1) microdroplet preparation; (2) microdroplet incubation and fermentation; (3) microdroplet SERS sorting. The specific steps are as follows:

(1) Microdroplet preparation: Freshly prepared bacterial solution was diluted with buffer to OD600 = 0.05 ~ 0.1 to be used as the aqueous phase, and the carrier oil phase was HFE-7500 Fluorinated Fluidics (3M) containing 2.0% (v/v) Pico-Surf surfactant (5% in HFE-7500, Sphere Fluidics). The diluted bacterial and oil phases were injected into the microfluidic droplet fabrication chip at a flow rate of 4 μL/min and 10 μL/min, respectively, to produce uniform droplets with a diameter of 30 μm. Buffer composition: fresh LB medium, 50 ug/mL kanamycin, 20 g/LPPA, 0.5 mM IPTG.

(2) Microdroplet incubation for fermentation: The resulting droplets containing cells were pooled in 50 mL anaerobic tubes and incubated anaerobically in a vertical incubator at 37 ℃ for 12 h without stirring.

(3) Microdroplet SERS sorting: microdroplets were detected in this thesis using a conical fiber-optic SERS probe. When the droplet flows through, the optical fiber automatically penetrates deep into the microdroplet for measurement, and measures one microdroplet at a time. During the measurement, the laser is injected from the optical fiber and detected at the end of the optical fiber, and then the light will return along the optical fiber automatically, and the optical fiber can be inserted into the droplet channel from different positions of the microfluidic chip according to the actual demand, and the end of the optical fiber amplifies the Raman signals, and the Raman signals are obtained for the screening through the characteristic Raman peaks. The sorting device is connected to the Raman detection system to control the sorting of microdroplets.

The collected droplets were re-injected into the sorting chip at a flow rate of 1 μL/min and separated using 20 μL/min of fluorinated oil (HFE-7500, 3M). The separated droplets were then detected by a fiber-optic SERS probe with an excitation light of 633 nm. Electrical deflection was used to direct target droplets with strong Raman signals into the sorting channel for collection, while droplets that did not meet expectations were discarded into the trash channel.

**Supplementary information**

Table S1 List of strains and plasmids used in this study

| Strains and plasmid | Relevant geneotype or description | Source or reference |
| --- | --- | --- |
| Strains |  |  |
| *E coli* MG1655 |  | Laboratory strain |
| *E coli* BL21(DE3) |  | Laboratory strain |
| BL21(DE3)-pET28a | BL21(DE3) harboring pET28a | Laboratory strain |
| CP303 | BL21(DE3) harboring pET28a-dldh-gldh | Laboratory strain |
| TB15 | MG1655 harboring pET28a- dldh^A174Q\V333R^ -R5-gldh | This work |
| TB10 | MG1655 harboring pET28a- dldh^A174Q\V333R^ -gldh | This work |
| TB00 | BL21(DE3) harboring pET28a- dldh^A174Q\V333R^ -gldh | This work |
| Plasmids |  |  |
| pET28a |  | Laboratory stock |
| pET28a-dldh | pET28a harboring the genes *dldh* | Laboratory stock |
| pET28a-dldh* | pET28a harboring the genes *dldh** | This work |
| pTg00 | pET28a harboring the genes *dldh** and *gldh* | This work |
| pTg01 | plasmid containing *dldh* gene and *gldh*, with RBS1 | This work |
| pTg02 | plasmid containing *dldh* gene and *gldh*, with RBS2 | This work |
| pTg03 | plasmid containing *dldh* gene and *gldh*, with RBS3 | This work |
| pTg04 | plasmid containing *dldh* gene and *gldh*, with RBS4 | This work |
| pTg05 | plasmid containing *dldh* gene and *gldh*, with RBS5 | This work |
| pTg06 | plasmid containing *dldh* gene and *gldh*, with RBS6 | This work |
| pTg07 | plasmid containing *dldh* gene and *gldh*, with RBS7 | This work |
| pTg08 | plasmid containing *dldh* gene and *gldh*, with RBS8 | This work |
| pTg09 | plasmid containing *dldh* gene and *gldh*, with RBS9 | This work |
| pTg10 | plasmid containing *dldh* gene and *gldh*, with RBS10 | This work |
| pTg11 | plasmid containing *dldh* gene and *gldh*, with RBS11 | This work |
| pTg12 | plasmid containing *dldh* gene and *gldh*, with RBS12 | This work |
| pTg13 | plasmid containing *dldh* gene and *gldh*, with RBS13 | This work |
| pTg14 | plasmid containing *dldh* gene and *gldh*, with RBS14 | This work |
| pTg15 | plasmid containing *dldh* gene and *gldh*, with RBS15 | This work |
| pTg16 | plasmid containing *dldh* gene and *gldh*, with RBS16 | This work |
| pTg17 | plasmid containing *dldh* gene and *gldh*, with RBS17 | This work |
| pTg18 | plasmid containing *dldh* gene and *gldh*, with RBS18 | This work |
| pTg19 | plasmid containing *dldh* gene and *gldh*, with RBS19 | This work |
| pTg20 | plasmid containing *dldh* gene and *gldh*, with RBS20 | This work |

Table S2 List of primers used in this study

| Primer name | Oligonucleotide sequence (5′→3′) ^a^ | Size (bp) |
| --- | --- | --- |
| dldh-F | CCGAATTCGAGCTCCGATGGCAAAAATTTACGCATACGGA | 40 |
| dldh-R | TAATGCGGTCCATGGAGCCACCACCGCCTTAACCAACCTTAACTGGG | 47 |
| gldh-F | AAGGTTGGTTAAGGCGGTGGTGGCTCCATGGACCGCATTATTCAATC | 47 |
| gldh-R | CCGCAAGCTTGTCGATTATTCCCACTCTTGCAGGAAACGC | 40 |
| pET28a-F | TCGACAAGCTTGCGGCCGCACTC | 23 |
| pET28a-R | CGGAGCTCGAATTCGGATCCGCGA | 24 |
| RBS1-F | AAGGTTGGTTAAAAAGATTAGAGTCATGGACCGCATTATTCAATC | 45 |
| RBS1-R | TAATGCGGTCCATGACTCTAATCTTTTTAACCAACCTTAACTGGG | 45 |
| RBS2-F | AAGGTTGGTTAAAAAGAGCTGAGCAATGGACCGCATTATTCAATC | 45 |
| RBS2-R | TAATGCGGTCCATTGCTCAGCTCTTTTTAACCAACCTTAACTGGG | 45 |
| RBS3-F | AAGGTTGGTTAAAAAGATTGGACGTATGGACCGCATTATTCAATC | 45 |
| RBS3-R | TAATGCGGTCCATACGTCCAATCTTTTTAACCAACCTTAACTGGG | 45 |
| RBS4-F | AAGGTTGGTTAAAAAGAGAGGAGCCATGGACCGCATTATTCAATC | 45 |
| RBS4-R | GATTGAATAATGCGGTCCATGGCTCCTCTCTTTTTAACCAACCTT | 45 |
| RBS5-F | AAGGTTGGTTAAAAAGAAGGGATACATGGACCGCATTATTCAATC | 45 |
| RBS5-R | TAATGCGGTCCATGTATCCCTTCTTTTTAACCAACCTTAACTGGG | 45 |
| RBS6-F | AAGGTTGGTTAAAAAGAGGGGACAAACTAGATGGACCGCATTATT | 45 |
| RBS6-R | TAATGCGGTCCATCTAGTTTGTCCCCTCTTTTTAACCAACCTTAACTG | 48 |
| RBS7-F | AAGGTTGGTTAAAAAGATCCGATGTACTAGATGGACCGCATTATT | 45 |
| RBS7-R | TAATGCGGTCCATCTAGTACATCGGATCTTTTTAACCAACCTTAACTG | 48 |
| RBS8-F | AAGGTTGGTTAAAAAGATTAGACAAACTAGATGGACCGCATTATT | 45 |
| RBS8-R | TAATGCGGTCCATCTAGTTTGTCTAATCTTTTTAACCAACCTTAACTG | 48 |
| RBS9-F | AAGGTTGGTTAAAAAGACGAGATATACTAGATGGACCGCATTATT | 45 |
| RBS9-R | TAATGCGGTCCATCTAGTATATCTCGTCTTTTTAACCAACCTTAACTG | 48 |
| RBS10-F | AAGGTTGGTTAAAAAGACTGGAGACACTAGATGGACCGCATTATT | 45 |
| RBS10-R | TAATGCGGTCCATCTAGTGTCTCCAGTCTTTTTAACCAACCTTAACTG | 48 |
| RBS11-F | TAAGGTTGGTTAAAAAGAGGCGATACACTAGATGGACCGCATTAT | 45 |
| RBS11-R | ATGCGGTCCATCTAGTGTATCGCCTCTTTTTAACCAACCTTAACTG | 46 |
| RBS12-F | TAAGGTTGGTTAAAAAGACATGAGTTACTAGATGGACCGCATTAT | 45 |
| RBS12-R | ATGCGGTCCATCTAGTAACTCATGTCTTTTTAACCAACCTTAACTG | 46 |
| RBS13-F | TAAGGTTGGTTAAAAAGATTTGATATACTAGATGGACCGCATTAT | 45 |
| RBS13-R | ATGCGGTCCATCTAGTATATCAAATCTTTTTAACCAACCTTAACTG | 46 |
| RBS14-F | TAAGGTTGGTTAAAAAGAGCCGACATACTAGATGGACCGCATTAT | 45 |
| RBS14-R | ATGCGGTCCATCTAGTATGTCGGCTCTTTTTAACCAACCTTAACTG | 46 |
| RBS15-F | TAAGGTTGGTTAAAAAGAGGTGACTCACTAGATGGACCGCATTAT | 45 |
| RBS15-R | ATGCGGTCCATCTAGTGAGTCACCTCTTTTTAACCAACCTTAACTG | 46 |
| RBS16-F | TAAGGTTGGTTAAAAAGACAGGATTAACTAGATGGACCGCATTAT | 45 |
| RBS16-R | ATGCGGTCCATCTAGTTAATCCTGTCTTTTTAACCAACCTTAACTG | 46 |
| RBS17-F | TAAGGTTGGTTAAAAAGACCGGAAATACTAGATGGACCGCATTAT | 45 |
| RBS17-R | ATGCGGTCCATCTAGTATTTCCGGTCTTTTTAACCAACCTTAACTG | 46 |
| RBS18-F | TAAGGTTGGTTAAAAAGACCGGAGACACTAGATGGACCGCATTAT | 45 |
| RBS18-R | ATGCGGTCCATCTAGTGTCTCCGGTCTTTTTAACCAACCTTAACTG | 46 |
| RBS19-F | TAAGGTTGGTTAAAAAGAAGGGACAGACTAGATGGACCGCATTAT | 45 |
| RBS19-R | ATGCGGTCCATCTAGTCTGTCCCTTCTTTTTAACCAACCTTAACTG | 46 |
| RBS20-F | TAAGGTTGGTTAAAAAGATAGGAGACACTAGATGGACCGCATTAT | 45 |
| RBS20-R | ATGCGGTCCATCTAGTGTCTCCTATCTTTTTAACCAACCTTAACTG | 46 |

Table S3 Random mutation PCR reaction system

| Name | Volume |
| --- | --- |
| Template Plasmid | 1 μL |
| 2×StarMut Random PCR Mix | 25 μL |
| Primer-F（10M） | 1 μL |
| Primer-R（10M） | 1 μL |
| StarMut Enhancer | 2.5 μL |
| Sterile Water | 19.5 μL |
| Total volume | 50 μL |

Table S4 Correlation between base mutation rate and amount of StarMut Enhancer

| reaction condition | 1 | 2 | 3 | 4 | 5 | 6 |
| --- | --- | --- | --- | --- | --- | --- |
| StarMut Enhancer | 0 | 1 | 2.5 | 3 | 5 | 10 |
| Number of mutated bases/kb | 0~1 | 0~3 | 0~4 | 0~4 | 1~6 | 3~10 |
| Average number of mutated bases/kb | 0.3 | 1.6 | 2.3 | 2.8 | 4.1 | 6.5 |


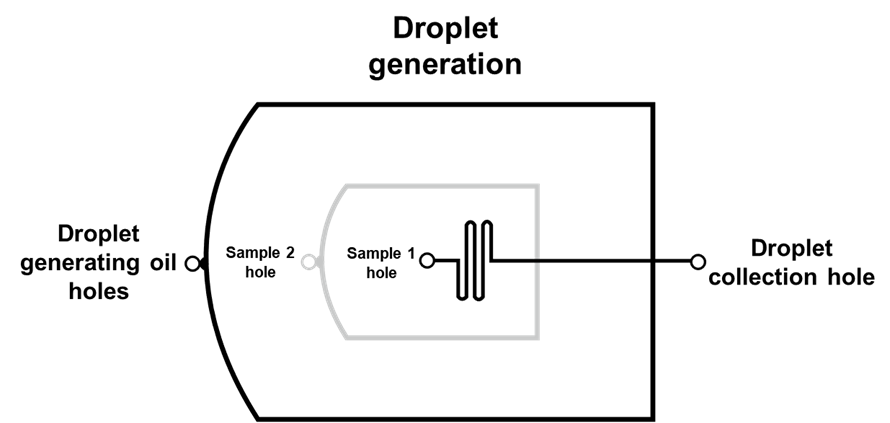


Figure S1: Schematic diagram of droplet generation chip


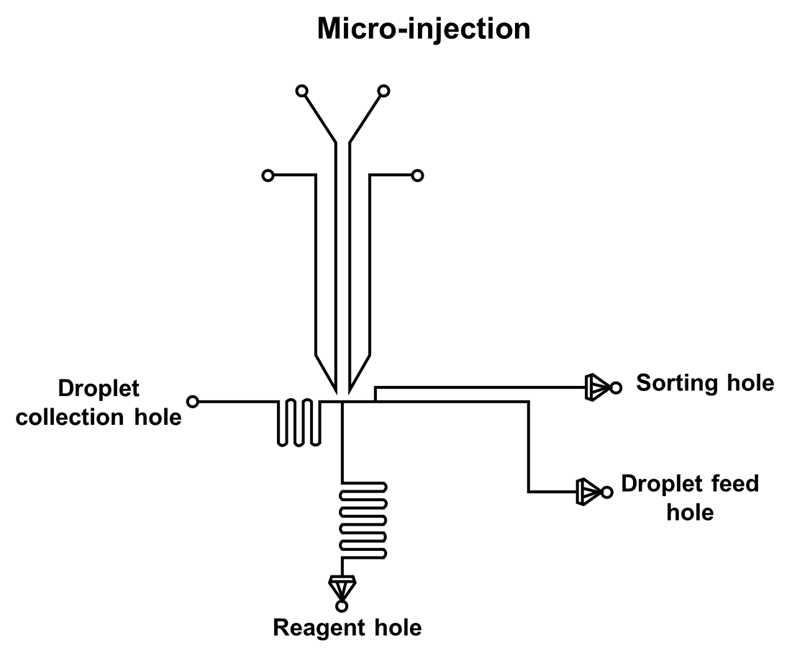


Figure S2: Schematic diagram of droplet microinjection chip


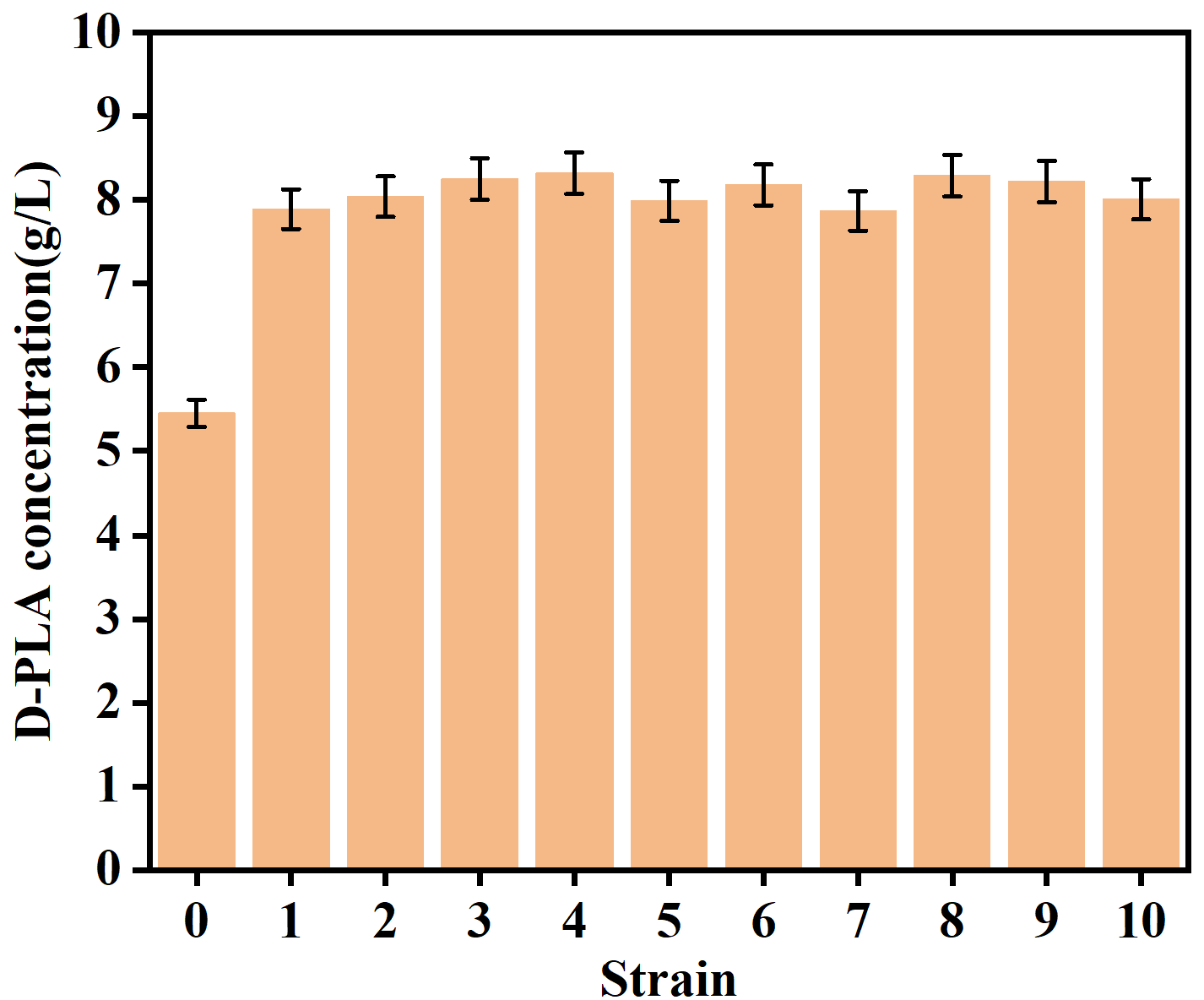


Figure S3: Comparison of yields of the mutant strain and the original strain


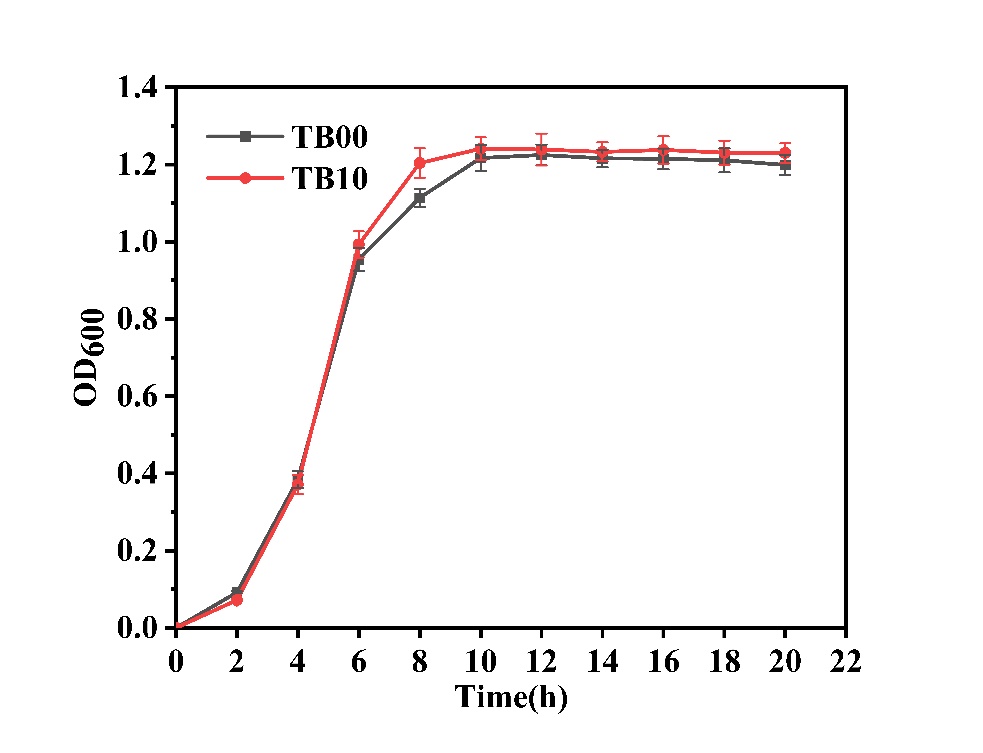


Figure S4: The cell growth of the strains TB00 (*E. coli* BL21(DE3) harboring the plasmid pTg00) and TB10 (*E. coli* MG1655 also carrying the plasmid pTg00)


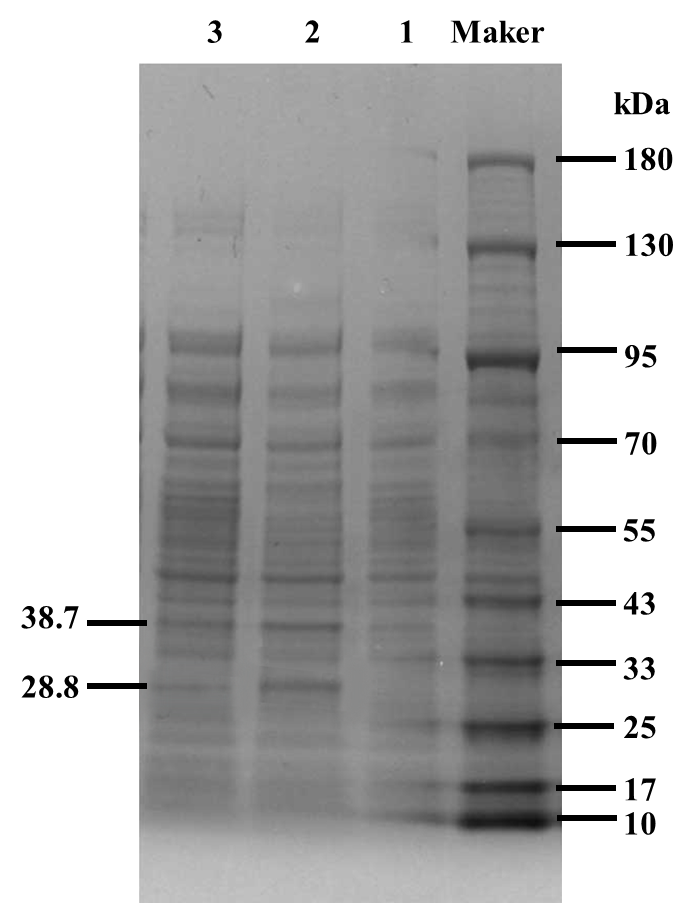


Figure S5: Lane 1 shows protein expression of BL21-pET28a strain. Lane 2 shows the protein expression of MG1655-pTg00 strain. Lane 3 shows protein expression of BL21-pTg00 strain


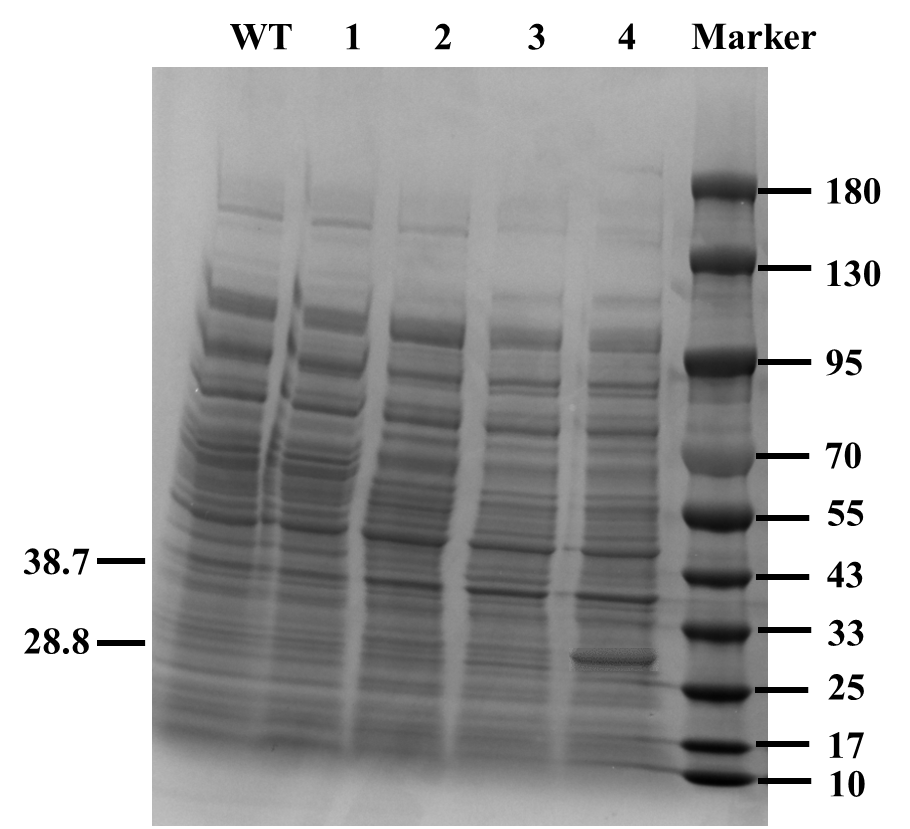


Figure S6: Comparison of protein expression after RBS sequence optimization. Lane 1, 2, 3, and 4 are strains with substitutions of R5, R10, R16, and R19, respectively. WT is the strain without RBS sequence substitution. Glycerol dehydrogenase protein size is 38.7 kDa and lactate dehydrogenase protein size is 28.8 kDa.


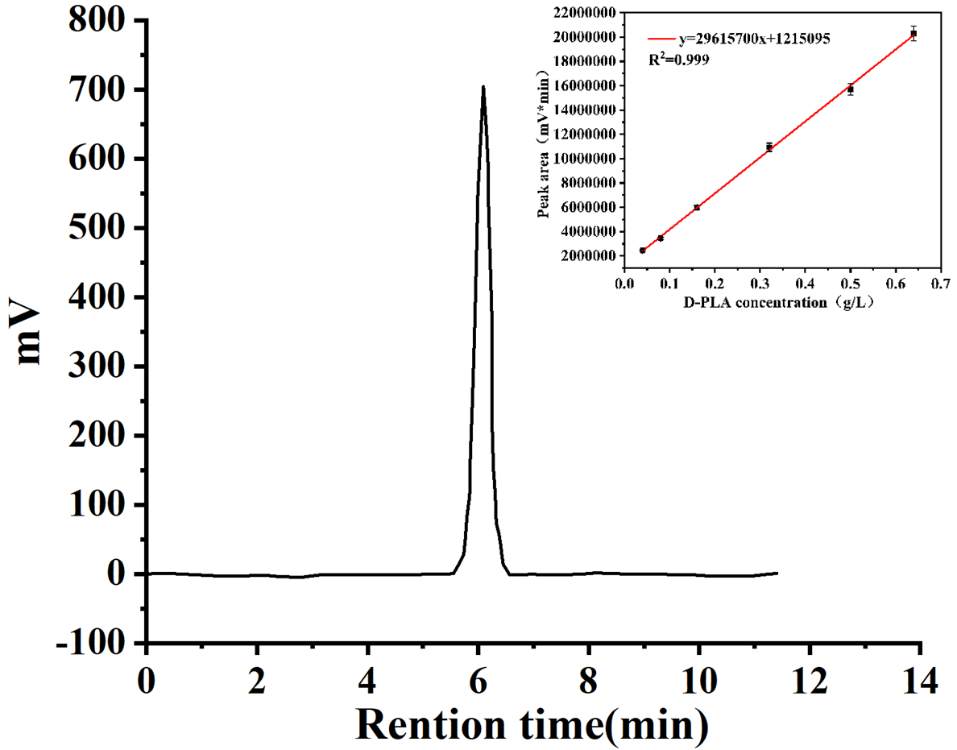


Figure S7: High performance liquid chromatogram of D-PLA standards


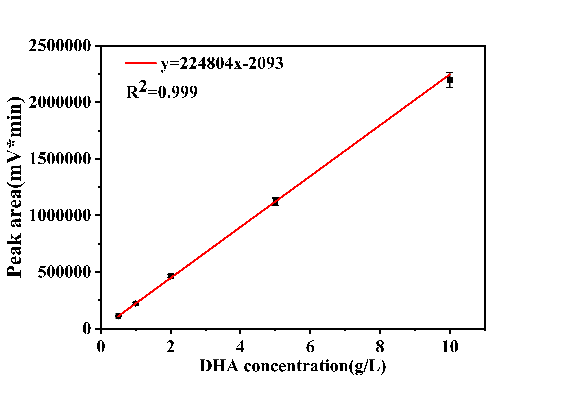




Figure S8: High performance liquid chromatogram of DHA standards
